# Diethyl 2,6-dimethyl-4-[5-(4-methyl­phen­yl)-1*H*-pyrazol-4-yl]-1,4-dihydro­pyridine-3,5-dicarboxyl­ate

**DOI:** 10.1107/S1600536812044649

**Published:** 2012-11-03

**Authors:** Arun M. Islor, Shridhar Malladi, Sandeep Telkar, Thomas Gerber, Eric Hosten, Richard Betz

**Affiliations:** a National Institute of Technology-Karnataka, Department of Chemistry, Medicinal Chemistry Laboratory, Surathkal, Mangalore 575 025, India; b Kuvempu University, Department of P.G. Studies and Research in Biotechnology and Bioinformatics, Jnanasahyadri–Karnataka, Shankaraghatta 577 451, India; c Nelson Mandela Metropolitan University, Summerstrand Campus, Department of Chemistry, University Way, Summerstrand, PO Box 77000, Port Elizabeth, 6031, South Africa

## Abstract

In the title compound, C_23_H_27_N_3_O_4_, the dihydro­pyridine ring adopts a ^1,4^ *B* conformation. Intra­molecular C—H⋯O contacts occur. In the crystal, N—H⋯O and N—H⋯N hydrogen bonds and C—H⋯N contacts connect the mol­ecules into strands along the *a-*axis direction.

## Related literature

For background to the biological and pharmaceutical importance of dihydro­pyridine compounds, see: Stout & Meyers (1982 ▶); Vijesh *et al.* (2011 ▶); Boecker & Guengerich (1986 ▶); Vo *et al.* (1995 ▶). For puckering analysis, see: Cremer & Pople (1975 ▶); Boeyens (1978 ▶). For graph-set analysis of hydrogen bonds, see: Etter *et al.* (1990 ▶); Bernstein *et al.* (1995 ▶).
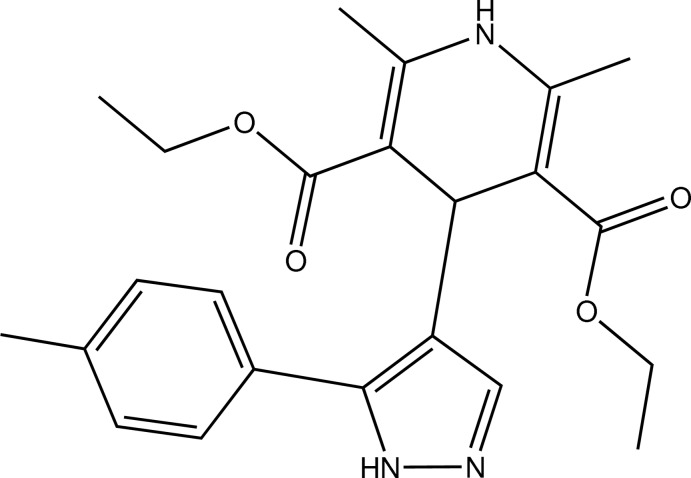


## Experimental

### 

#### Crystal data

C_23_H_27_N_3_O_4_
*M* *_r_* = 409.48
Triclinic, 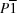

*a* = 8.5905 (2) Å
*b* = 10.8253 (3) Å
*c* = 11.3702 (3) Å
α = 91.021 (1)°
β = 97.922 (1)°
γ = 93.445 (1)°
*V* = 1045.02 (5) Å^3^
*Z* = 2
Mo *K*α radiation
μ = 0.09 mm^−1^
*T* = 200 K
0.36 × 0.31 × 0.16 mm

#### Data collection

Bruker APEXII CCD diffractometer
Absorption correction: multi-scan (*SADABS*; Bruker, 2008 ▶) *T*_min_ = 0.969, *T*_max_ = 0.986
18073 measured reflections
4983 independent reflections
4313 reflections with *I* > 2σ(*I*)
*R*_int_ = 0.017

#### Refinement

*R*[*F* ^2^ > 2σ(*F* ^2^)] = 0.041
*wR*(*F* ^2^) = 0.113
*S* = 1.05
4983 reflections
284 parameters
H atoms treated by a mixture of independent and constrained refinement
Δρ_max_ = 0.33 e Å^−3^
Δρ_min_ = −0.21 e Å^−3^

### 

Data collection: *APEX2* (Bruker, 2010 ▶); cell refinement: *SAINT* (Bruker, 2010 ▶); data reduction: *SAINT*; program(s) used to solve structure: *SHELXS97* (Sheldrick, 2008 ▶); program(s) used to refine structure: *SHELXL97* (Sheldrick, 2008 ▶); molecular graphics: *ORTEP-3* (Farrugia, 2012 ▶) and *Mercury* (Macrae *et al.*, 2008 ▶); software used to prepare material for publication: *SHELXL97* and *PLATON* (Spek, 2009 ▶).

## Supplementary Material

Crystal structure: contains datablock(s) I, global. DOI: 10.1107/S1600536812044649/hg5264sup1.cif

Supplementary material file. DOI: 10.1107/S1600536812044649/hg5264Isup2.cdx

Structure factors: contains datablock(s) I. DOI: 10.1107/S1600536812044649/hg5264Isup3.hkl

Supplementary material file. DOI: 10.1107/S1600536812044649/hg5264Isup4.cml

Additional supplementary materials:  crystallographic information; 3D view; checkCIF report

## Figures and Tables

**Table 1:** Hydrogen-bond geometry (Å, °)

*D*—H⋯*A*	*D*—H	H⋯*A*	*D*⋯*A*	*D*—H⋯*A*
N11—H11*B*⋯N21^i^	0.890 (17)	2.200 (17)	3.0352 (14)	156.2 (14)
N22—H22⋯O3^ii^	0.892 (17)	2.066 (17)	2.9580 (13)	178.6 (15)
C2—H2*B*⋯N21^iii^	0.98	2.52	3.4223 (16)	153
C36—H36⋯O1	0.95	2.30	3.2428 (17)	173

Symmetry codes: (i) 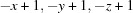; (ii) 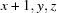; (iii) 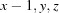.

**Fig. 1.**
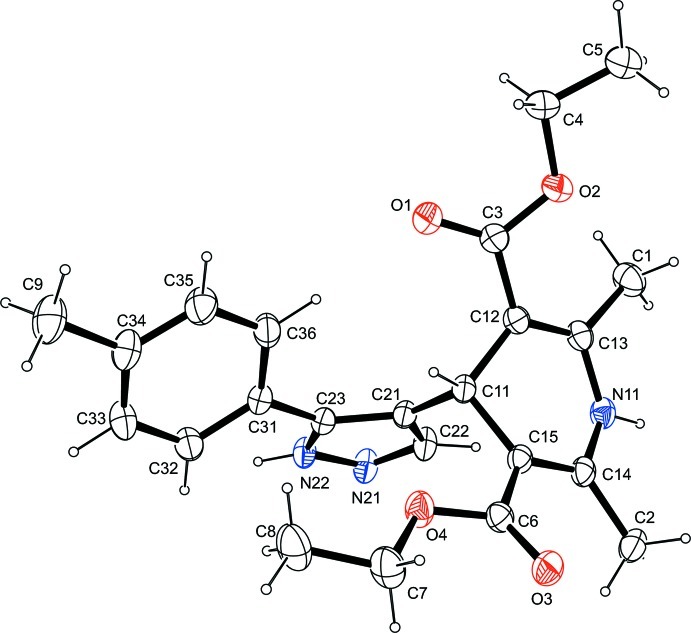
The molecular structure of the title compound, with atom labels and anisotropic displacement ellipsoids (drawn at 50% probability level).

**Fig. 2.**
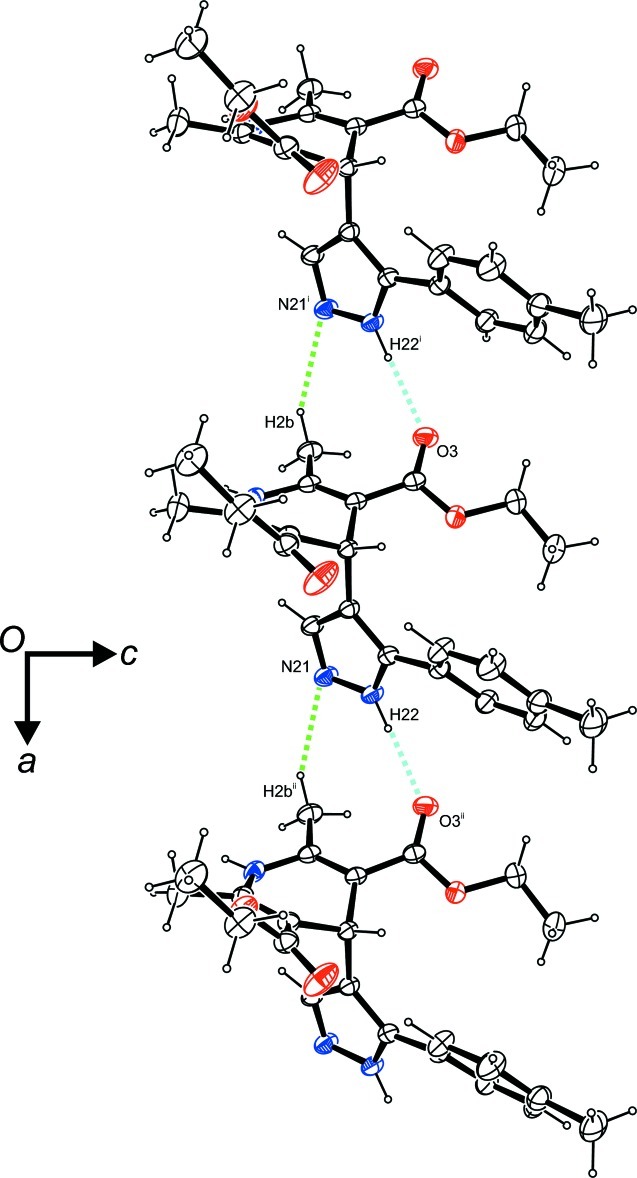
Intermolecular contacts, viewed along [0 1 0]. For reasons of clarity, only a selection of contacts is shown. Blue dashed lines indicate classical hydrogen bonds of the N–H···O type, green dashed lines denote C–H···N contacts. Symmetry operators: ix - 1, y, z; iix + 1, y, z.

**Fig. 3.**
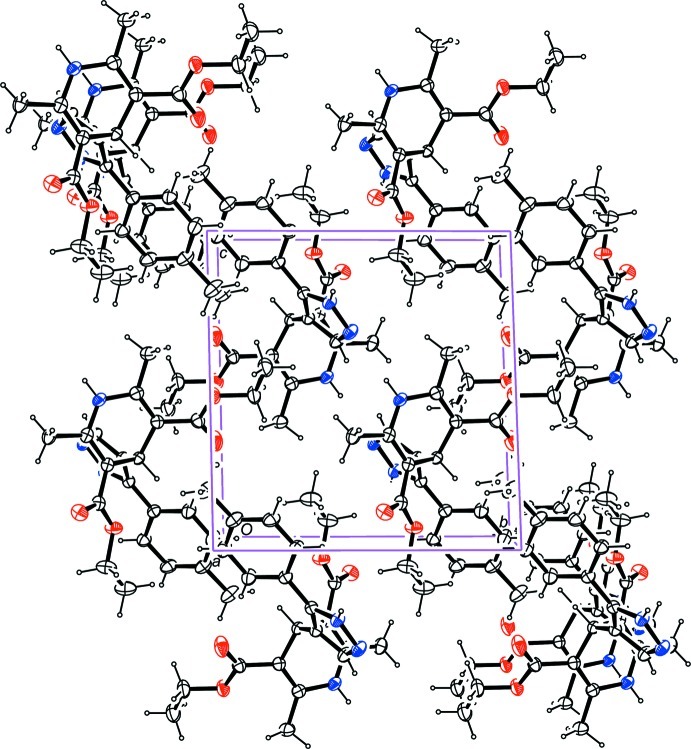
Molecular packing of the title compound, viewed along [-1 0 0] (anisotropic displacement ellipsoids drawn at 50% probability level).

## supplementary crystallographic information

### Comment

The 1,4-dihydropyridine scaffold (DHP) represents a heterocyclic unit of remarkable pharmacological activity (Stout & Meyers, 1982) and is found to exhibit various biological activities (Vijesh *et al.*, 2011). Several DHP-inspired compounds are already used clinically as calcium channel blockers for the treatment of cardiovascular diseases, such as Nifedipine and Nitrendipine – used for the treatment of hypertension and angina pectoris –, Nisoldipine – a potent vasodilator – and Nimodipine – selective agent for targeting cerebral vasculature – to name but a few (Boecker & Guengerich, 1986). A number of DHP derivatives are discussed as potential drugs for the treatment of congestive heart failure (Vo *et al.*, 1995). Motivated by the promising pharmaceutical activities of 1,4-dihydropyridines, the title compound was synthesized to study its crystal structure.

The molecule is a pyrazole derivative featuring a phenyl as well as a dihydropyridine-derived substituent. While the small puckering amplitude precludes a conformational analysis of the five-membered heterocycle, the dihyropyridine ring adopts a ^1,4^*B* (^N11,C11^*B*) conformation (Cremer & Pople, 1975; Boeyens, 1978). The least-squares planes defined by the intracyclic atoms of the phenyl group as well as the five-membered heterocycle, respectively, enclose an angle of 33.03 (7) °. The plane defined by the atoms of the dihydropyridine core is almost planar (r.m.s. of all fitted atoms = 0.1726 Å), with the flap carbon atom and the nitrogen atom deviating most from the plane by 0.270 (1) Å and 0.199 (1) Å, respectively (Fig. 1).

In the crystal, classical intermolecular hydrogen bonds of the N–H···N as well as the N–H···O type are present. Furthermore, C–H···N contacts and C–H···O contacts whose range falls by more than 0.1 Å below the sum of van-der-Waals radii of the corresponding atoms are observed. The C–H···N contacts are supported by one of the hydrogen atoms of a methyl group directly bonded to the dihydropyridine ring and one of the nitrogen atoms of the pyrazole moiety. The intramolecular C–H···O contact is apparent between one of the hydrogen atoms of the phenyl group in *ortho* position to the pyrazole ring and one of the *sp*^2^ hybridized oxygen atoms. Metrical parameters as well as information about the symmetry of these contacts are summarized in Table 1. In total, the C–H···N contacts as well as the N–H···O-type hydrogen bonds form antidromic chains of molecules that are extended to strands along the crystallographic *a* axis by the N–H···N-type hydrogen bonds. In terms of graph-set analysis (Etter *et al.*, 1990; Bernstein *et al.*, 1995), the descriptor for the classical hydrogen bonds is *C*^1^_1_(8)*R*^2^_2_(16) on the unary level. The C–H supported contacts necessitate a *S*(9)*C*^1^_1_(8) descriptor on the same level. The shortest intercentroid distance between two aromatic systems was measured at 4.7738 (8) Å (Fig. 2).

The packing of the title compound in the crystal structure is shown in Figure 3.

### Experimental

3-(4-methylphenyl)-1*H*-pyrazole-4-carbaldehyde(0.187 g, 1.0 mmol), ethylacetoacetate (0.26 g, 2.0 mmol) and ammonium acetate (0.092 g, 1.2 mmol) in ethanol (7 ml) were refluxed in an oil bath for 5 h. After completion of the reaction, the mixture was concentrated and poured into crushed ice. The precipitated product was filtered and washed with water. The resulting solid was recrystallized from ethanol:water (*v*:*v* = 1:1), yield: 0.33 g (80.68%).

### Refinement

Carbon-bound H atoms were placed in calculated positions (C–H 0.95 Å for aromatic carbon atoms, C–H 0.99 Å for methylene groups and C–H 1.00 Å for the methine group) and were included in the refinement in the riding model approximation, with *U*(H) set to 1.2*U*_eq_(C). The H atoms of the methyl groups were allowed to rotate with a fixed angle around the C–C bond to best fit the experimental electron density (HFIX 137 in the *SHELX* program suite (Sheldrick, 2008), with *U*(H) set to 1.5*U*_eq_(C). Both nitrogen-bound H atoms were located on a difference Fourier map and refined freely.

### Crystal data

**Table:**

C_23_H_27_N_3_O_4_	*Z* = 2
*M**_r_* = 409.48	*F*(000) = 436
Triclinic, *P*1	*D*_x_ = 1.301 Mg m^−^^3^
Hall symbol: -P 1	Melting point = 471–473 K
*a* = 8.5905 (2) Å	Mo *K*α radiation, λ = 0.71073 Å
*b* = 10.8253 (3) Å	Cell parameters from 9960 reflections
*c* = 11.3702 (3) Å	θ = 2.7–28.3°
α = 91.021 (1)°	µ = 0.09 mm^−^^1^
β = 97.922 (1)°	*T* = 200 K
γ = 93.445 (1)°	Block, yellow
*V* = 1045.02 (5) Å^3^	0.36 × 0.31 × 0.16 mm

### Data collection

**Table:**

Bruker APEXII CCD diffractometer	4983 independent reflections
Radiation source: fine-focus sealed tube	4313 reflections with *I* > 2σ(*I*)
Graphite monochromator	*R*_int_ = 0.017
φ and ω scans	θ_max_ = 28.0°, θ_min_ = 2.4°
Absorption correction: multi-scan (*SADABS*; Bruker, 2008)	*h* = −11→11
*T*_min_ = 0.969, *T*_max_ = 0.986	*k* = −14→14
18073 measured reflections	*l* = −15→14

### Refinement

**Table:**

Refinement on *F*^2^	Primary atom site location: structure-invariant direct methods
Least-squares matrix: full	Secondary atom site location: difference Fourier map
*R*[*F*^2^ > 2σ(*F*^2^)] = 0.041	Hydrogen site location: inferred from neighbouring sites
*wR*(*F*^2^) = 0.113	H atoms treated by a mixture of independent and constrained refinement
*S* = 1.05	*w* = 1/[σ^2^(*F*_o_^2^) + (0.0515*P*)^2^ + 0.4147*P*] where *P* = (*F*_o_^2^ + 2*F*_c_^2^)/3
4983 reflections	(Δ/σ)_max_ < 0.001
284 parameters	Δρ_max_ = 0.33 e Å^−^^3^
0 restraints	Δρ_min_ = −0.21 e Å^−^^3^

### Fractional atomic coordinates and isotropic or equivalent isotropic displacement parameters (Å^2^)

**Table:**

	*x*	*y*	*z*	*U*_iso_*/*U*_eq_	
O1	0.45908 (13)	0.00208 (9)	0.66938 (10)	0.0463 (3)	
O2	0.25873 (11)	−0.00254 (8)	0.52185 (9)	0.0327 (2)	
O3	0.15357 (10)	0.44024 (9)	0.88752 (8)	0.0317 (2)	
O4	0.37379 (10)	0.34282 (9)	0.94459 (8)	0.0304 (2)	
N11	0.25151 (12)	0.37638 (9)	0.53483 (9)	0.0245 (2)	
H11B	0.2162 (19)	0.4218 (15)	0.4735 (15)	0.034 (4)*	
N21	0.76600 (12)	0.46269 (10)	0.68190 (10)	0.0280 (2)	
N22	0.82226 (12)	0.39623 (9)	0.77631 (9)	0.0242 (2)	
H22	0.923 (2)	0.4087 (15)	0.8088 (14)	0.033 (4)*	
C1	0.24030 (17)	0.21083 (13)	0.38718 (11)	0.0343 (3)	
H1A	0.1319	0.1744	0.3764	0.051*	
H1B	0.3115	0.1484	0.3679	0.051*	
H1C	0.2478	0.2810	0.3345	0.051*	
C2	0.15003 (15)	0.53629 (12)	0.64804 (12)	0.0297 (3)	
H2A	0.1726	0.5759	0.7273	0.045*	
H2B	0.0363	0.5174	0.6282	0.045*	
H2C	0.1865	0.5924	0.5894	0.045*	
C3	0.36694 (14)	0.05624 (11)	0.60280 (11)	0.0266 (2)	
C4	0.25461 (18)	−0.13674 (12)	0.51954 (14)	0.0362 (3)	
H4A	0.2425	−0.1689	0.5990	0.043*	
H4B	0.3530	−0.1658	0.4957	0.043*	
C5	0.11687 (18)	−0.17966 (13)	0.43120 (14)	0.0403 (3)	
H5A	0.0216	−0.1448	0.4528	0.060*	
H5B	0.1041	−0.2702	0.4305	0.060*	
H5C	0.1342	−0.1521	0.3521	0.060*	
C6	0.26466 (13)	0.38529 (11)	0.86200 (11)	0.0237 (2)	
C7	0.35707 (17)	0.36804 (16)	1.06793 (12)	0.0378 (3)	
H7A	0.2562	0.3296	1.0866	0.045*	
H7B	0.3589	0.4584	1.0837	0.045*	
C8	0.49288 (19)	0.31395 (17)	1.14178 (13)	0.0443 (4)	
H8A	0.4903	0.3340	1.2258	0.066*	
H8B	0.5918	0.3486	1.1184	0.066*	
H8C	0.4855	0.2238	1.1294	0.066*	
C9	0.90941 (19)	−0.02184 (15)	1.18484 (14)	0.0431 (4)	
H9A	0.8981	0.0137	1.2629	0.065*	
H9B	1.0204	−0.0360	1.1817	0.065*	
H9C	0.8465	−0.1007	1.1719	0.065*	
C11	0.41373 (12)	0.25920 (10)	0.72388 (10)	0.0203 (2)	
H11	0.4153	0.1989	0.7895	0.024*	
C12	0.35319 (13)	0.19111 (11)	0.60642 (10)	0.0226 (2)	
C13	0.28586 (14)	0.25489 (11)	0.51391 (11)	0.0244 (2)	
C14	0.23363 (13)	0.41889 (11)	0.64688 (11)	0.0228 (2)	
C15	0.29660 (13)	0.35556 (10)	0.74185 (10)	0.0210 (2)	
C21	0.57727 (13)	0.32159 (10)	0.72757 (10)	0.0211 (2)	
C22	0.61806 (14)	0.41725 (12)	0.65375 (11)	0.0265 (3)	
H22A	0.5473	0.4465	0.5902	0.032*	
C23	0.71422 (13)	0.31013 (10)	0.80674 (10)	0.0214 (2)	
C31	0.75666 (13)	0.22762 (11)	0.90584 (10)	0.0231 (2)	
C32	0.86603 (15)	0.26894 (12)	1.00279 (11)	0.0282 (3)	
H32	0.9084	0.3523	1.0074	0.034*	
C33	0.91302 (16)	0.18844 (13)	1.09262 (12)	0.0327 (3)	
H33	0.9883	0.2177	1.1578	0.039*	
C34	0.85291 (15)	0.06632 (13)	1.08966 (12)	0.0321 (3)	
C35	0.74117 (17)	0.02728 (13)	0.99461 (13)	0.0357 (3)	
H35	0.6959	−0.0551	0.9921	0.043*	
C36	0.69364 (15)	0.10560 (12)	0.90293 (12)	0.0311 (3)	
H36	0.6180	0.0760	0.8381	0.037*	

### Atomic displacement parameters (Å^2^)

**Table:**

	*U*^11^	*U*^22^	*U*^33^	*U*^12^	*U*^13^	*U*^23^
O1	0.0470 (6)	0.0281 (5)	0.0564 (7)	0.0101 (4)	−0.0218 (5)	−0.0044 (5)
O2	0.0351 (5)	0.0228 (4)	0.0369 (5)	0.0006 (4)	−0.0064 (4)	−0.0024 (4)
O3	0.0226 (4)	0.0383 (5)	0.0348 (5)	0.0059 (4)	0.0047 (4)	−0.0034 (4)
O4	0.0269 (4)	0.0430 (5)	0.0215 (4)	0.0090 (4)	0.0015 (3)	−0.0001 (4)
N11	0.0230 (5)	0.0253 (5)	0.0242 (5)	0.0011 (4)	−0.0010 (4)	0.0067 (4)
N21	0.0209 (5)	0.0310 (5)	0.0316 (5)	0.0012 (4)	0.0004 (4)	0.0109 (4)
N22	0.0178 (5)	0.0265 (5)	0.0273 (5)	0.0015 (4)	−0.0012 (4)	0.0056 (4)
C1	0.0409 (7)	0.0360 (7)	0.0239 (6)	−0.0007 (6)	−0.0016 (5)	0.0017 (5)
C2	0.0244 (6)	0.0273 (6)	0.0373 (7)	0.0063 (5)	0.0012 (5)	0.0064 (5)
C3	0.0243 (6)	0.0256 (6)	0.0292 (6)	0.0018 (4)	0.0018 (5)	−0.0019 (5)
C4	0.0398 (7)	0.0222 (6)	0.0439 (8)	0.0016 (5)	−0.0033 (6)	−0.0025 (5)
C5	0.0424 (8)	0.0287 (7)	0.0462 (8)	−0.0004 (6)	−0.0042 (6)	−0.0033 (6)
C6	0.0189 (5)	0.0236 (5)	0.0277 (6)	−0.0015 (4)	0.0016 (4)	0.0002 (4)
C7	0.0341 (7)	0.0567 (9)	0.0229 (6)	0.0055 (6)	0.0049 (5)	−0.0031 (6)
C8	0.0413 (8)	0.0642 (10)	0.0255 (7)	0.0036 (7)	−0.0025 (6)	0.0007 (6)
C9	0.0408 (8)	0.0478 (9)	0.0400 (8)	0.0045 (7)	0.0001 (6)	0.0213 (7)
C11	0.0176 (5)	0.0212 (5)	0.0216 (5)	0.0016 (4)	0.0004 (4)	0.0029 (4)
C12	0.0199 (5)	0.0229 (5)	0.0245 (5)	−0.0003 (4)	0.0022 (4)	0.0000 (4)
C13	0.0209 (5)	0.0265 (6)	0.0248 (6)	−0.0025 (4)	0.0013 (4)	0.0012 (4)
C14	0.0158 (5)	0.0230 (5)	0.0287 (6)	−0.0008 (4)	0.0010 (4)	0.0031 (4)
C15	0.0164 (5)	0.0212 (5)	0.0251 (5)	0.0002 (4)	0.0016 (4)	0.0017 (4)
C21	0.0182 (5)	0.0224 (5)	0.0223 (5)	0.0031 (4)	0.0007 (4)	0.0019 (4)
C22	0.0195 (5)	0.0304 (6)	0.0287 (6)	0.0020 (4)	−0.0006 (4)	0.0089 (5)
C23	0.0188 (5)	0.0221 (5)	0.0229 (5)	0.0031 (4)	0.0012 (4)	0.0009 (4)
C31	0.0195 (5)	0.0258 (6)	0.0239 (5)	0.0046 (4)	0.0011 (4)	0.0033 (4)
C32	0.0265 (6)	0.0293 (6)	0.0272 (6)	−0.0008 (5)	−0.0008 (5)	0.0033 (5)
C33	0.0287 (6)	0.0409 (7)	0.0262 (6)	0.0007 (5)	−0.0047 (5)	0.0060 (5)
C34	0.0272 (6)	0.0384 (7)	0.0311 (6)	0.0052 (5)	0.0023 (5)	0.0127 (5)
C35	0.0344 (7)	0.0284 (6)	0.0419 (8)	−0.0012 (5)	−0.0030 (6)	0.0101 (6)
C36	0.0275 (6)	0.0286 (6)	0.0339 (7)	0.0007 (5)	−0.0070 (5)	0.0045 (5)

### Geometric parameters (Å, º)

**Table:**

O1—C3	1.2041 (16)	C7—H7A	0.9900
O2—C3	1.3373 (15)	C7—H7B	0.9900
O2—C4	1.4508 (15)	C8—H8A	0.9800
O3—C6	1.2195 (15)	C8—H8B	0.9800
O4—C6	1.3403 (14)	C8—H8C	0.9800
O4—C7	1.4521 (15)	C9—C34	1.5050 (18)
N11—C14	1.3785 (16)	C9—H9A	0.9800
N11—C13	1.3886 (16)	C9—H9B	0.9800
N11—H11B	0.890 (17)	C9—H9C	0.9800
N21—C22	1.3292 (15)	C11—C21	1.5167 (15)
N21—N22	1.3527 (14)	C11—C15	1.5223 (15)
N22—C23	1.3586 (15)	C11—C12	1.5247 (15)
N22—H22	0.892 (17)	C11—H11	1.0000
C1—C13	1.5015 (17)	C12—C13	1.3505 (16)
C1—H1A	0.9800	C14—C15	1.3559 (16)
C1—H1B	0.9800	C21—C23	1.3926 (15)
C1—H1C	0.9800	C21—C22	1.4029 (16)
C2—C14	1.4977 (16)	C22—H22A	0.9500
C2—H2A	0.9800	C23—C31	1.4704 (15)
C2—H2B	0.9800	C31—C32	1.3945 (17)
C2—H2C	0.9800	C31—C36	1.3952 (17)
C3—C12	1.4720 (16)	C32—C33	1.3877 (17)
C4—C5	1.488 (2)	C32—H32	0.9500
C4—H4A	0.9900	C33—C34	1.388 (2)
C4—H4B	0.9900	C33—H33	0.9500
C5—H5A	0.9800	C34—C35	1.3845 (19)
C5—H5B	0.9800	C35—C36	1.3885 (18)
C5—H5C	0.9800	C35—H35	0.9500
C6—C15	1.4641 (16)	C36—H36	0.9500
C7—C8	1.496 (2)		
			
C3—O2—C4	116.55 (10)	C34—C9—H9A	109.5
C6—O4—C7	116.94 (10)	C34—C9—H9B	109.5
C14—N11—C13	121.46 (10)	H9A—C9—H9B	109.5
C14—N11—H11B	117.7 (10)	C34—C9—H9C	109.5
C13—N11—H11B	119.0 (10)	H9A—C9—H9C	109.5
C22—N21—N22	103.83 (9)	H9B—C9—H9C	109.5
N21—N22—C23	112.96 (10)	C21—C11—C15	109.87 (9)
N21—N22—H22	119.8 (10)	C21—C11—C12	113.53 (9)
C23—N22—H22	127.1 (10)	C15—C11—C12	106.87 (9)
C13—C1—H1A	109.5	C21—C11—H11	108.8
C13—C1—H1B	109.5	C15—C11—H11	108.8
H1A—C1—H1B	109.5	C12—C11—H11	108.8
C13—C1—H1C	109.5	C13—C12—C3	123.65 (11)
H1A—C1—H1C	109.5	C13—C12—C11	119.48 (10)
H1B—C1—H1C	109.5	C3—C12—C11	116.82 (10)
C14—C2—H2A	109.5	C12—C13—N11	118.14 (11)
C14—C2—H2B	109.5	C12—C13—C1	128.04 (11)
H2A—C2—H2B	109.5	N11—C13—C1	113.82 (11)
C14—C2—H2C	109.5	C15—C14—N11	118.45 (10)
H2A—C2—H2C	109.5	C15—C14—C2	127.45 (11)
H2B—C2—H2C	109.5	N11—C14—C2	114.04 (10)
O1—C3—O2	122.52 (11)	C14—C15—C6	121.38 (10)
O1—C3—C12	124.48 (11)	C14—C15—C11	118.89 (10)
O2—C3—C12	112.88 (10)	C6—C15—C11	119.52 (10)
O2—C4—C5	106.23 (11)	C23—C21—C22	103.87 (10)
O2—C4—H4A	110.5	C23—C21—C11	130.71 (10)
C5—C4—H4A	110.5	C22—C21—C11	125.13 (10)
O2—C4—H4B	110.5	N21—C22—C21	113.03 (10)
C5—C4—H4B	110.5	N21—C22—H22A	123.5
H4A—C4—H4B	108.7	C21—C22—H22A	123.5
C4—C5—H5A	109.5	N22—C23—C21	106.30 (10)
C4—C5—H5B	109.5	N22—C23—C31	120.22 (10)
H5A—C5—H5B	109.5	C21—C23—C31	133.47 (11)
C4—C5—H5C	109.5	C32—C31—C36	118.69 (11)
H5A—C5—H5C	109.5	C32—C31—C23	120.25 (11)
H5B—C5—H5C	109.5	C36—C31—C23	121.01 (11)
O3—C6—O4	122.45 (11)	C33—C32—C31	120.06 (12)
O3—C6—C15	126.12 (11)	C33—C32—H32	120.0
O4—C6—C15	111.42 (10)	C31—C32—H32	120.0
O4—C7—C8	106.79 (11)	C32—C33—C34	121.70 (12)
O4—C7—H7A	110.4	C32—C33—H33	119.2
C8—C7—H7A	110.4	C34—C33—H33	119.2
O4—C7—H7B	110.4	C35—C34—C33	117.73 (12)
C8—C7—H7B	110.4	C35—C34—C9	120.89 (13)
H7A—C7—H7B	108.6	C33—C34—C9	121.37 (13)
C7—C8—H8A	109.5	C34—C35—C36	121.64 (13)
C7—C8—H8B	109.5	C34—C35—H35	119.2
H8A—C8—H8B	109.5	C36—C35—H35	119.2
C7—C8—H8C	109.5	C35—C36—C31	120.15 (12)
H8A—C8—H8C	109.5	C35—C36—H36	119.9
H8B—C8—H8C	109.5	C31—C36—H36	119.9
			
C22—N21—N22—C23	0.64 (14)	C21—C11—C15—C14	83.76 (12)
C4—O2—C3—O1	1.44 (19)	C12—C11—C15—C14	−39.82 (13)
C4—O2—C3—C12	−174.64 (11)	C21—C11—C15—C6	−91.09 (12)
C3—O2—C4—C5	174.86 (12)	C12—C11—C15—C6	145.33 (10)
C7—O4—C6—O3	−1.67 (18)	C15—C11—C21—C23	116.11 (13)
C7—O4—C6—C15	179.04 (11)	C12—C11—C21—C23	−124.30 (13)
C6—O4—C7—C8	−179.02 (12)	C15—C11—C21—C22	−56.77 (15)
O1—C3—C12—C13	159.81 (14)	C12—C11—C21—C22	62.82 (15)
O2—C3—C12—C13	−24.20 (17)	N22—N21—C22—C21	−0.49 (14)
O1—C3—C12—C11	−22.62 (18)	C23—C21—C22—N21	0.18 (14)
O2—C3—C12—C11	153.37 (10)	C11—C21—C22—N21	174.63 (11)
C21—C11—C12—C13	−83.11 (13)	N21—N22—C23—C21	−0.54 (14)
C15—C11—C12—C13	38.18 (14)	N21—N22—C23—C31	178.60 (10)
C21—C11—C12—C3	99.21 (12)	C22—C21—C23—N22	0.21 (13)
C15—C11—C12—C3	−139.50 (10)	C11—C21—C23—N22	−173.80 (11)
C3—C12—C13—N11	167.37 (11)	C22—C21—C23—C31	−178.77 (12)
C11—C12—C13—N11	−10.14 (16)	C11—C21—C23—C31	7.2 (2)
C3—C12—C13—C1	−12.1 (2)	N22—C23—C31—C32	32.05 (17)
C11—C12—C13—C1	170.34 (11)	C21—C23—C31—C32	−149.08 (13)
C14—N11—C13—C12	−22.13 (16)	N22—C23—C31—C36	−145.42 (12)
C14—N11—C13—C1	157.45 (11)	C21—C23—C31—C36	33.4 (2)
C13—N11—C14—C15	20.38 (16)	C36—C31—C32—C33	1.51 (19)
C13—N11—C14—C2	−162.15 (10)	C23—C31—C32—C33	−176.02 (11)
N11—C14—C15—C6	−171.78 (10)	C31—C32—C33—C34	−0.5 (2)
C2—C14—C15—C6	11.14 (18)	C32—C33—C34—C35	−1.2 (2)
N11—C14—C15—C11	13.47 (15)	C32—C33—C34—C9	177.43 (13)
C2—C14—C15—C11	−163.61 (11)	C33—C34—C35—C36	2.0 (2)
O3—C6—C15—C14	21.00 (18)	C9—C34—C35—C36	−176.65 (14)
O4—C6—C15—C14	−159.74 (11)	C34—C35—C36—C31	−1.1 (2)
O3—C6—C15—C11	−164.28 (11)	C32—C31—C36—C35	−0.75 (19)
O4—C6—C15—C11	14.98 (14)	C23—C31—C36—C35	176.77 (12)

### Hydrogen-bond geometry (Å, º)

**Table:**

*D*—H···*A*	*D*—H	H···*A*	*D*···*A*	*D*—H···*A*
N11—H11*B*···N21^i^	0.890 (17)	2.200 (17)	3.0352 (14)	156.2 (14)
N22—H22···O3^ii^	0.892 (17)	2.066 (17)	2.9580 (13)	178.6 (15)
C2—H2*B*···N21^iii^	0.98	2.52	3.4223 (16)	153
C36—H36···O1	0.95	2.30	3.2428 (17)	173

Symmetry codes: (i) −*x*+1, −*y*+1, −*z*+1; (ii) *x*+1, *y*, *z*; (iii) *x*−1, *y*, *z*.
